# A dashboard for monitoring preventive measures in response to COVID-19 outbreak in the Democratic Republic of Congo

**DOI:** 10.1186/s41182-020-00262-3

**Published:** 2020-08-26

**Authors:** Patient Mijiriro Wimba, Jacques-Aimé Bazeboso, Philippe Bianga Katchunga, Léon Tshilolo, Benjamin Longo-Mbenza, Muriel Rabilloud, Philippe Vanhems, Jean Iwaz, Jean-François Étard, René Écochard

**Affiliations:** 1 Université Officielle de Bukavu, Bukavu, Democratic Republic of Congo; 2 Cliniques Universitaires de Bukavu, Bukavu, Democratic Republic of Congo; 3 Université de Lyon, Lyon, France; 4 Université de Kinshasa, Kinshasa, Democratic Republic of Congo; 5 Équipe Biostatistique Santé, Laboratoire de Biométrie et Biologie Évolutive, CNRS UMR 5558, Villeurbanne, France; 6 Service de Biostatistique-Bioinformatique, Pôle Santé Publique, Hospices Civils de Lyon, Lyon, France; 7 Service d‘Hygiène Hospitalière, Épidémiologie, Infectiovigilance et Prévention, Hospices Civils de Lyon, Lyon, France; 8 Laboratoire des Pathogènes Émergents, Centre International de Recherche en Infectiologie (CIRI), Université de Lyon, Lyon, France; 9 TransVIHMI (Institut de Recherche pour le Développement, IRD UMI 233; Institut National de la Santé et de la Recherche Médicale, INSERM U 1175; Université de Montpellier), Montpellier, France; 10 EpiGreen, Paris, France

**Keywords:** COVID-19, Dashboard, Low-income countries, Urban environment

## Abstract

**Background:**

In most health areas, an information system is necessary for an effective fight against COVID-19. Current methods for surveillance of diseases with epidemic potential do not include monitoring the adherence to preventive measures. Furthermore, modern data collection methods depend often on technologies (e.g., cameras or drones) that are hardly available in low-income countries. Simpler solutions could be just as effective.

**Methods:**

A dashboard was used over a whole week to monitor preventive measures in Bukavu (DRC) by mid-May 2020. It was designed to collect from street passers-by information on the adherence to barrier measures, the level of awareness of these measures, the opinion on their usefulness, and the health status of people in the households.

**Results:**

Creating a dashboard and collecting the necessary data proved feasible. The use of barrier measures was very limited and that of masks practically nil despite repeated recommendations from the health authorities. The end of each day was the worst moment due to clearly insufficient distancing. Barrier measures were significantly more used in areas where they were best known and most acknowledged. At the time of the study, there were few sick people and only rare severe cases were attributed to COVID-19.

**Conclusions:**

Creating COVID-19 situation dashboards in limited-resource metropoles is feasible. They give real-time access to data that help fight the epidemic. The findings of this pilot study call for a rapid community awareness actions to back national media-based prevention campaigns.

## Introduction

On May 11, 2020, the Ministry of Health of the Democratic Republic of Congo (DRC) had already recorded a little more than one thousand confirmed cases of COVID-19 [1]. Most of these cases were diagnosed in Kinshasa though cases were also identified in various provinces, especially in South Kivu. Since then, the health authorities of DRC established national and provincial committees to contain the disease outbreak. To adapt their decisions to the epidemic spread, these committees have to be regularly informed about changes in the spread of the disease through the population and the degree to which the population adheres to preventive barrier measures.

Most DRC provinces are used to deal with health crises and, when the COVID-19 crisis broke out, the DRC was about to declare the end of the Ebola epidemic that erupted in 2018 in North Kivu. The experience of past epidemics being likely to orient responses to new epidemics [2], the population of Bukavu (Eastern DRC, South Kivu) was informed about the onset of the COVID-19 pandemic since April 2020 and called to wear masks and use measures to protect people over 60 [3].

The management of a health crisis has to follow a process that requires accurate data about the extent of the epidemic, the changes in morbidity and mortality rates, the adoption by the population of preventive measures, and the understanding and perceptions of the population [4]. Furthermore, each new crisis is an opportunity to improve previous procedures, especially in terms of data collection. For example, since March 2020, COSMO study (COVID-19 Snapshot MOnitoring) in Germany has required weekly updates about interactions between risk perception, awareness, and disinformation [5]. Besides, many other epidemic-specific initiatives have been taken worldwide, including the creation of dashboards to collect data to monitor the extent of the epidemic [6]. The methods of data collection should be adapted to each country or even each region.

The present article reports on a pilot experience for the creation of a dashboard in limited-resource settings. It was conducted in the streets of Bukavu by mid-May 2020. It describes (i) the process of data collection and analysis; (ii) the findings regarding the use of barrier measures, the level of awareness of these measures among the passers-by, and the opinions of the latter on the measures’ usefulness; and (iii) some information about the health status of people of their households.

## Materials and methods

### Study setting and procedures

The study was carried out over a single week (May 14 to May 20, 2020) in Bukavu, a city of one million inhabitants. Five out of the 37 health areas of Bukavu were selected for the study because they represent the diversity of Bukavu’s population: Muhungu Diocésain, Ceca-40 Nguba, Maria, Neema, and Burhiba. These areas included 165,549 inhabitants.

The data were collected by ten observers recruited among the young physicians of Bukavu. These volunteer observers had to test the feasibility of a data collection method (or dashboard) before its implementation in wider parts of the city. They were invited to attend four meetings for consultation and training of which two were held before data collection and two during data collection.

In each of the five health areas, three typical streets were chosen among the busiest, the medium busy, and the quietest streets of the area. The choices were made after discussions with the observers who know well their city and the activities along its streets.

The observers had to visit the streets five times daily. The approximate times of the visits (local time ± 1 h) were 8 AM (designated as “early morning”), 11 AM (“morning”), 12:30 PM (“noon”), 5 PM (“end of the day”), and 7 PM (“early evening”).

The observers had to score the use of barrier measures in the streets and interview some passers-by (Table 1). The data were stored in a software application downloaded to the observer’s smartphones. Examining the use of barrier measures included scoring three indicators: (i) street population density, (ii) physical distancing, and (iii) masking (Table S1). The indicators were rated on a Likert scale: 0 for the most pejorative to 3 for the most favorable regarding the fight against the epidemic (Table S1).

**Table 1:** Content of the questionnaire given to passers-by in the streets of Bukavu, DRC, May 2020

Questions and suggested answers	Score	Number (%)*
The interviewed person		
Women		101 (37.0%)
Age		24.5 (15; 76)
Place of residence		
Within 1 km	2	147 (53.5%)
Farther than 1 km in the city	1	114 (4.1%)
Out of the city	0	14 (5.1%)
Knowledge about the barrier measures		
Keep a minimum distance		
Well known and understood	3	158 (57.0%)
Fairly known	2	71 (25.6%)
Poorly known or understood	1	35 (12.6%)
Not known	0	13 (4.7%)
Never touch a person		
Well known and understood	3	156 (56.3%)
Fairly known	2	81 (29.2%)
Poorly known or understood	1	33 (11.9%)
Not known	0	7 (2.5%)
Cough into the elbow		
Well known and understood	3	147 (53.2%)
Fairly known	2	66 (23.9%)
Poorly known or understood	1	51 (18.5%)
Not known	0	12 (4.3%)
Wash hands frequently with soap and water or hydroalcoholic gel		
Well known and understood	3	191 (69.2%)
Fairly known	2	57 (20.7%)
Poorly known or understood	1	25 (9.1%)
Not known	0	3 (1%)
Wear a mask		
Well known and understood	3	172 (61.9%)
Fairly known	2	68 (24.5%)
Poorly known or understood	1	32 (11.5%)
Not known	0	6 (2.2%)
Wash frequently the mask		
Well known and understood	3	171 (62.6%)
Fairly known	2	56 (20.5%)
Poorly known or understood	1	35 (12.8%)
Not known	0	11 (4.0%)
Opinion about the usefulness of the barrier measures		
Very useful	3	180 (64.7%)
Fairly useful	2	62 (22.3%)
Poorly useful	1	23 (8.3%)
Useless	0	13 (4.7%)
Health status of persons within the household		
Number of persons living in the household		6 (1; 20)
Number of sick persons living in the household		20.9
Probable link between one or more diseases with the current epidemic		
None		270 (98.2%)
At least one		4 (1.5%)
Two or more		1 (0.3%)
Number of death within the past 30 days		4.8
Probable link between one or more deaths with the current epidemic		
None		273 (100%)
At least one		--
Two or more		--

*Other values are either median (min; max) or number per 1000 inhabitants

On each street visit, each observer had to take at least one photo for later checks of the ratings. Each photo had to be taken from the same spot with the same view angle. It was automatically geolocated, dated, and uploaded to the secure server of the study. Later, two independent assessors had to analyze the photos and rate the indicators. The agreements between the three ratings were later analyzed.

Each observer had to interview two passers-by immediately or at his/her next visit. Each interview was geolocated, dated, and saved on the server of the study. It included three parts: (i) awareness of the barrier measures, (ii) opinion about their usefulness, and (iii) the health status of people in the household. The answer to each question was also rated on a similar Likert scale: 0 for the most pejorative answer (ignorance or uselessness) to 3 for the most favorable answer (awareness and understanding) regarding the fight against the epidemic (Table 1).

No personal data were collected, except for sex and age. The photos were immediately destructed after assessors’ checks.

### Statistical analysis

The mean scores for street population density, distancing, and masking were calculated over the whole city at each of the five times of each observation day.

The distributions of the scores on the items of the questionnaire were tabulated as numbers and percentages. A mean score was calculated for each response modality.

Regarding the health status of people in the household, the study considered a ratio of the number of sick people to the number of all people in the household. The result was later expressed per one thousand inhabitants.

Differences between health areas were assessed using Pearson’s chi-square test. The mean scores (with their 95% confidence intervals) for street population density, distancing, masking, awareness of barrier measures, and their usefulness were calculated per health area.

Correlation coefficients of Spearman were estimated to quantify the relationships between five variables: street population density, distancing, masking, awareness of barriers, and opinion about barriers. The results were tabulated and expressed graphically. The agreements between observers’ and assessors’ ratings were estimated using weighted Kappa coefficients.

All data were entered and stored on a dedicated website. All statistical analyses were carried out with R software (R Core Team, 2019; R: a language and environment for statistical computing; R Foundation for Statistical Computing, Vienna, Austria; URL http://www.R-project.org). Significance was set at *p* < 0.05.

## Results

### Creation of the dashboard

Within each of the five health areas, three streets were daily visited. This resulted in 105 observation days (i.e., 5 × 3 × 7). The number of daily visits was 3 over 98 days, 2 visits over 6 days, and 4 visits over one day; this resulted in 310 visits (i.e., (98 × 3) + (6 × 2) + 4). Field staffs used to spend nearly 20 min at each street visit (median, 18 min; lower quartile, 9 min; upper quartile, 29 min).

### Statements regarding passers-by density, distancing, and masking

**Fig. 1:**
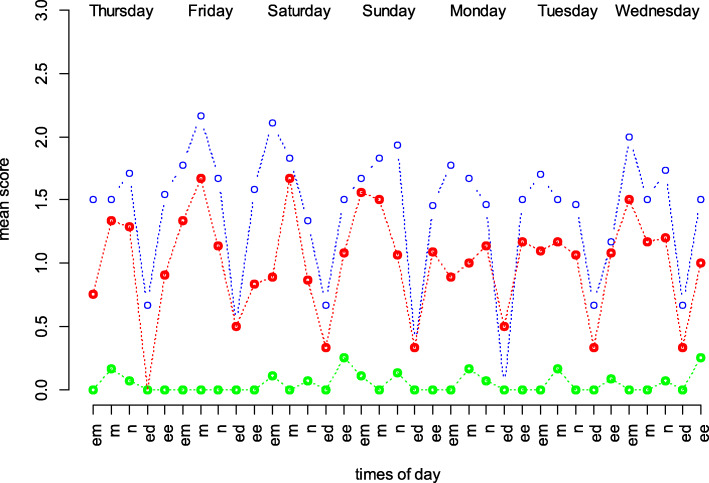
Mean scores of street population density (blue), distancing (red), and masking (green) at various times of the day on each of the 7 days of the study (em, early morning; m, morning; n, noon; ed, end of the day; ee, early evening). The study was carried out between Thursday, 14 May and Wednesday, 20 May, 2020

The first impressive finding was the wide difference in passers-by densities according to the time of the day. Figure 1 shows the daily periodic oscillations of the mean density score and the mean distancing score. There were no significant differences in each of these mean scores between weekdays and weekends.

A low street population density (i.e., high mean density score) is favorable to prevention against contamination. The mean density scores (blue points) were close to value 2 at noon (low density) but had much lower values during the rest of the day. This mean score dropped to its minimum value (0.5) at the end of the day, which would indicate that it was ranging between 0 (very high density) and 1 (relatively high density).

Distancing reflected the proportion of passers-by distant by more than 1 m. The red line in Fig. 1 shows the daily changes in the mean score for distancing. This line is overall parallel to the line that shows passers-by density: it oscillates between 1.5 at noon and 0.5 at the end of the day. The former value means that the distancing scores were generally equal to 1 (distance often < 1 m) or to 2 (distance often > 1 m). Values at 0.5 seen at the end of the day mean that the scores were either equal to zero (distance always < 1 m) or to one (distance often < 1 m).

Masking was anecdotal whatever the time of the day; the mean score for masking was often close to zero (no passer-by wearing a mask). Over all 310 street visits, masks were seen only on 16 photos (5%) of which 15 were showing only 1 out of 3 passers-by wearing a mask. Only one visit showed all passers-by with masks.

### Awareness of barrier measures and opinion about their usefulness

The observers interviewed 279 passers-by. Of these, 2 out of 3 were men, 1 out of 2 was aged ≤ 24.5 years, and 1 out of 3 aged > 30 years. Only 5% were strangers to Bukavu, whereas 50% lived within 1 km of the street where they were interviewed (Table 1).

The awareness of the barrier measures was rather satisfactory in at least 50% of interviewed passers-by (min 53% for coughing in the elbow; max 69% for handwashing). Roughly, up to 12% were hardly or poorly aware of these measures. Finally, 87% of interviewed passers-by believed the measures were “very useful” or “fairly useful,” whereas 13% considered them “poorly useful” or “useless” (Table 1).

The number of sick persons per household on each day of the survey was rather low, nearly 2%. In 2% of the declarations, the interviewed passers-by attributed the health problem to COVID-19. In the households of the passers-by, one out of 200 persons had died within the last month. No death was attributed to COVID-19.

### Differences between health areas

The mean score for street population density varied significantly between health areas (*p* < 0.001); it ranged between 1.03 (95% CI 0.85; 1.21) and 2.29 (2.16; 2.42). The mean score for distancing varied also significantly between health areas (*p* < 0.001); it ranged between 0.48 (0.34; 0.63) and 1.45 (1.23; 1.67). There was no significant difference between health areas regarding masking.

The mean score for awareness of the barrier measures varied significantly between areas (*p* < 0.001); it ranged between 1.92 (1.75; 2.09) and 2.91 (2.86; 2.95).

The mean score relative to the opinion of interviewed passers-by regarding the usefulness of the barrier measures varied also significantly between areas (*p* < 0.001); it ranged between 1.84 (1.59; 2.09) and 2.83 (2.67; 2.98).

### Correlations between the main variables of the questionnaire

**Fig. 2:**
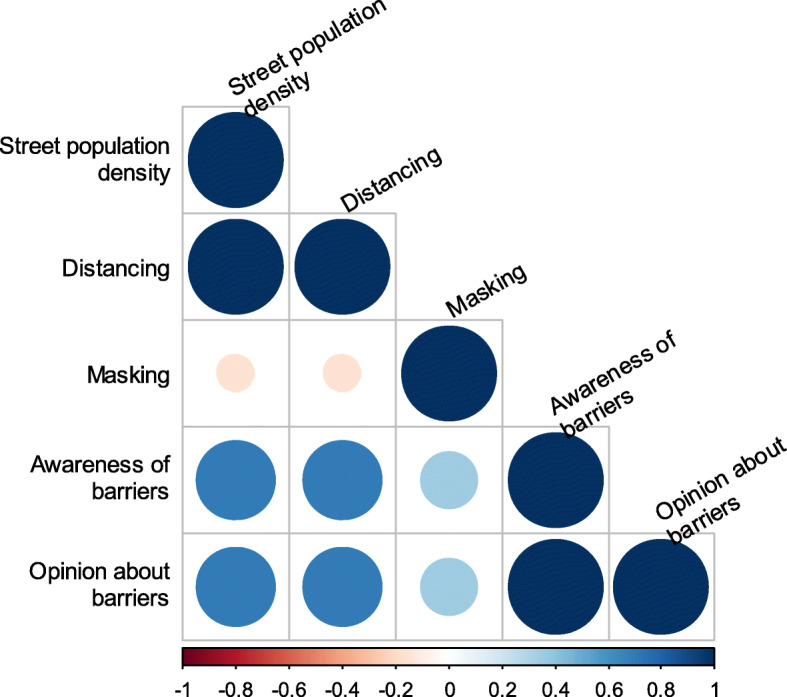
Correlation matrix between the main variables of the questionnaire. The colored scale shows the values of Spearman correlation coefficients. Blue points correspond to positive correlations and pink ones to negative correlations. The size of each point is proportional to the strength of correlation

Figure 2 and Table S2 show the correlation matrix between these variables.

The areas with low street population density and appropriate distancing were also the areas where the interviewees were aware of the barrier measures and had a positive opinion about their usefulness.

Unexpectedly and inexplicably, there was no significant correlation between masking and low street population density or appropriate distancing.

The areas where the interviewees were aware of the barrier measures and had a positive opinion about their usefulness were also the areas where masking was more frequent, but the latter correlation was not found statistically significant.

### Agreement between field observers and photo assessors

Kappa coefficients for agreement regarding street population density and distancing were 0.49 (95% CI 0.42; 0.56) and 0.24 (95% CI 0.15; 0.33) which represent, respectively, a moderate agreement and a fair agreement according to the classification of Landis and Koch [7]. No coefficient could be calculated for masking because of the very low number of photos showing people wearing masks.

## Discussion

Within the setting of this study, creating a dashboard and collecting the necessary data proved feasible. The observers reported a limited use of barrier measures and practically no use of masks despite repeated recommendations from the health authorities, good awareness of these measures, and acknowledgment of their usefulness. At the end of each day, the situation used to worsen given the nearly lack of distancing. A non-negligible part of the population (roughly, 30 to 50%) was still ignoring the barrier measures or did not fully acknowledge their usefulness (up to 13%). These measures were more frequently used in areas where they were the best known and the most acknowledged as useful. Generally, awareness of barrier measures was satisfactorily correlated with supportive opinion on them but, regretfully, weakly correlated with masking. At the time of the study, the number of sick people was low and only rare severe cases were attributed to COVID-19.

A poor compliance with barrier measures despite a good awareness of their usefulness was often seen elsewhere and in previous health crises. However, in this study, a particular attention should be paid to the place of the study. Bukavu is located in Kivu province whose North was the epicenter of a recent Ebola epidemic. This may explain the antagonism between knowledge and compliance:( i) the people had a good awareness of barrier measures because most measures are common to Ebola virus disease and COVID-19, and (ii) they had a poor compliance with masking because they ignored or poorly integrated the need to protect themselves against a possible contamination via contact with apparently healthy persons (healthy carriers). In a document on the measures to apply during an Ebola epidemic, the WHO stated: “Only people who are sick can spread Ebola disease to others” [8]. With COVID-19, the situation is different because the virus is transmissible for several days before the appearance of symptoms [9]. The people of Bukavu may probably not have a clear idea of that difference and, thus, may not have been inclined to comply with barrier measures when they met persons with no symptoms. This recalls one condition of success of a crisis management: a good understanding of the risk [10]. Furthermore, it is already well established that barrier measures should be proof-based, simple, adaptable, rapidly put to use, easy to use, and cost-efficient. The latter condition is obviously crucial in countries where the population is poor or vulnerable [11]. In fact, measures that lack coherence are unsuitable for the social setting or proposed with few empathies that may lead to suspicion and fear [12].

In most African metropoles, the end of the day is the privileged moment for conviviality and informal trade (which is often necessary for survival). This time is also undoubtedly the most difficult moment for controlling disease transmission. Currently, in such a context, masking seems to be the best way to control the spread of the disease [13]. In other countries (e.g., Taiwan), masking played a major role in stopping the epidemic [14]. However, announcing a compulsory mask-wearing measure was not sufficient to generalize the use of masks. We finally believe that masking-recommendation dissemination, explanation, and iteration by various authorities, at various levels, by all possible media and by local field initiatives may ultimately help achieve the desired behavior change.

The study found that a small but not negligible part of the population was still ignoring the barrier measures or did not acknowledge their usefulness. Despite its small number, this part might include superspreaders whose behavior may have a substantial impact on the disease dynamics [15]. Educational programs targeting poorly educated or marginal people seem necessary for a successful fight against the epidemic.

The present study showed that barrier measures were more frequently used in health areas where they were the best known and the most acknowledged but its design did not allow establishing a cause-effect relationship. Yet, in other contexts, it has been established that awareness and acceptance of barrier measures have positive effects on people’s behavior [16].

In addition, this study showed that national media-based campaigns are insufficient for reaching high levels of compliance with barrier measures. This demonstrated the need for local interventions to improve understanding, help behavior change, and promote adherence to new hygiene routines. To obtain behavior change, it seems essential to reach a good understanding of the information [17]. Local community interventions at the level of city districts seem necessary and feasible. In fact, information through the media does not seem to improve population adherence to barrier measures. Erroneous interpretations were still present in the study region where successive epidemics have called for distinct recommendations and attitudes.

The agreements between observers and assessors regarding passers-by density or distancing were not satisfactory. The assessors found it difficult to comment on the photos. Estimating the distances between passers-by on photos was not an easy task. In more favorable economic contexts, the use of drones would be a better option because of the possibilities of measuring distances, counting passers-by, and identifying masked ones [18]. Another possibility is to rely on observers’ statements only or use two independent observers on a random sample of streets but this entails a loss of documentation through photography.

The success of this pilot study in an African metropole demonstrates the feasibility of creating dashboards for monitoring COVID-19 in limited-resource urban areas. This was possible using available human resources and minimal technical resources. Indeed, in such areas, a number of young physicians who do not have paid employments are willing to accept temporary jobs and the nearly global access to the Internet on personal smartphones helps data entry and management. Besides, data management and analysis costs were markedly reduced with the use of a web application able to perform real-time calculations that may be made readily available to the health authorities without extra costs. These assets, especially the low costs, would give any limited-resource metropole reasonable abilities to create a dashboard. During the COVID-19 crisis, a great number of institutions [19] and cities in the world have created dashboards and appreciated their utilities. KAP-type (knowledge-attitude-practice) questionnaires (such as the one used in Bukavu) seem to be well suited to the African setting [20]. In fact, collecting information about the awareness of barrier measures, the opinion of the population, and the epidemiology of the disease in terms of morbidity and mortality is a major asset in the management of future health crises similar to the COVID-19 pandemic [12].

## Conclusion

Creating dashboards to monitor the implementation of preventive measures is feasible in limited-resource metropoles. These dashboards give real-time access to data that help fight against the spread of an epidemic. The findings of the present pilot study call for fast local community awareness actions to support national prevention campaigns. It is hoped the study’s encouraging results will give a chance for a small part of the budget devoted to fighting the COVID-19 pandemic in a limited-resource setting to be directed toward creating this type of dashboards, especially in densely populated cities.

## Supplementary information

**Additional file 1: Table S1.** Statements and scores used by the observers in the streets of Bukavu, DRC, May 2020. **Table S2.** Spearman correlation coefficient between the main variables of the survey.

## Data Availability

The datasets used and/or analyzed during the current study are available from the corresponding author on reasonable request.

## Abbreviations

CI: Confidence interval
COSMO: COVID-19 Snapshot MOnitoring
COVID: Coronavirus disease
DRC: Democratic Republic of Congo
KAP: Knowledge-attitude-practice
WHO: World Health Organization
