# Supplementary material for: A dashboard for monitoring preventive measures in response to COVID-19 outbreak in the Democratic Republic of Congo
Source: Trop Med Health. 2020 Aug 26;48:74. doi: 10.1186/s41182-020-00262-3 (PMC7447591; doi:10.1186/s41182-020-00262-3)
Supplement: Supplementary file 1 — Additional file 1: Table S1. Statements and scores used by the observers in the streets of Bukavu, DRC, May 2020. Table S2. Spearman correlation coefficient between the main variables of the survey. [file 41182_2020_262_MOESM1_ESM.docx]

**Table S1 – Statements and scores used by the observers in the streets of Bukavu, DRC, May 2020.**

| **Statement** | **Score** |
| --- | --- |
| *Street population density* |  |
| Very low | 3 |
| Low | 2 |
| High | 1 |
| Very high | 0 |
| *Physical distancing* |  |
| Always >1 m | 3 |
| Often >1 m | 2 |
| Often <1 m | 1 |
| Nearly always <1 m | 0 |
| *Masking* |  |
| All people masked | 3 |
| More than 2/3 | 2 |
| Nearly 1/3 | 1 |
| Very few or no masking | 0 |

**Table S2 – Spearman correlation coefficients between the main variables of the survey.**

|  | Street population density | Distancing | Masking | Awareness of barrier measures | Opinion about barrier measures |
| --- | --- | --- | --- | --- | --- |
| Street population density | 1 | 1 | -0.15 | 0.7 | 0.7 |
| Distancing | 1 | 1 | -0.15 | 0.7 | 0.7 |
| Masking | -0.15 | -0.15 | 1 | 0.36 | 0.36 |
| Awareness of barrier measures | 0.7 | 0.7 | 0.36 | 1 | 1 |
| Opinion about barrier measures | 0.7 | 0.7 | 0.36 | 1 | 1 |
